# ECIS technology reveals that monocytes isolated by CD14^+ve^ selection mediate greater loss of BBB integrity than untouched monocytes, which occurs to a greater extent with IL-1β activated endothelium in comparison to TNFα

**DOI:** 10.1371/journal.pone.0180267

**Published:** 2017-07-21

**Authors:** Dan Ting Kho, Rebecca Johnson, Laverne Robilliard, Elyce du Mez, Julie McIntosh, Simon J. O’Carroll, Catherine E. Angel, E. Scott Graham

**Affiliations:** 1 Centre for Brain Research, School of Medical Sciences, Faculty of Medical and Health Sciences, University of Auckland, Auckland, New Zealand; 2 Department of Pharmacology and Clinical Pharmacology, School of Medical Sciences, Faculty of Medical and Health Sciences, University of Auckland, Auckland, New Zealand; 3 School of Biological Sciences, Faculty of Science, University of Auckland, Auckalnd, New Zealand; 4 Department of Anatomy and Medical Imaging, School of Medical Sciences, Faculty of Medical and Health Sciences, University of Auckland, Auckland, New Zealand; Institute of Neurology (Edinger-Institute), GERMANY

## Abstract

**Background:**

We have previously shown that TNFα and IL-1β differentially regulate the inflammatory phenotype of human brain endothelial cells (hCMVECs). In this regard, IL-1β treatment was considerably more potent than TNFα at increasing expression of inflammatory chemokines and leukocyte adhesion molecules. We therefore hypothesised that interaction of the hCMVECs with human monocytes would also be dependent on the activation status of the endothelium. Therefore, the primary aim of this study was to assess whether brain endothelial cells activated by IL-1β or TNFα differed in their interaction with monocytes.

**Methods:**

Monocyte interaction was measured using the real time, label-free impedance based ECIS technology, to evaluate endothelial barrier integrity during monocyte attachment and transendothelial migration.

**Results:**

ECIS technology revealed that there was a greater loss of barrier integrity with IL-1β activation and this loss lasted for longer. This was expected and consistent with our hypothesis. However, more striking and concerning was the observation that the method of monocyte enrichment greatly influenced the extent of endothelial barrier compromise. Importantly, we observed that positively isolated monocytes (CD14^+ve^) caused greater reduction in barrier resistance, than the negatively selected monocytes (untouched). Analysis of the isolated monocyte populations revealed that the CD14^+ve^ isolation consistently yields highly pure monocytes (>92%), whereas the untouched isolation was much more variable, yielding ~70% enrichment on average. These two enrichment methods were compared as it was thought that the presence of non-classical CD16^hi^ monocytes in the untouched enrichment may mediate greater compromise than the classical CD14^hi^ monocytes. This however, was not the case and these observations raise a number of important considerations pertaining to the enrichment strategy, which are essential for generating reliable and consistent data.

**Conclusions:**

We conclude that IL-1β and TNFα differentially influence monocyte interaction with brain endothelial cells and moreover, the enrichment method also influences the monocyte response as revealed using ECIS technology.

## Introduction

The blood brain barrier (BBB) is a selectively permeable physical barrier composed of vascular endothelial cells, pericytes and astrocytes. The brain is highly vascularised and the BBB endothelial cells tightly regulate transport of nutrients, secretion of waste/toxins, and exclude entry to bacterial and viral particles (reviewed by [1]). In addition, the BBB endothelium is critical in controlling the migration of immune cells between the blood and the brain [2]. Brain vascular endothelial cells are characterized by the high density of tight junction and adherens junction complexes, thus providing brain endothelial cells with a low paracellular permeability to cells and solutes (reviewed by [3]). The tightness of the BBB is vital in maintaining the brain’s immune specialised status.

During neuroinflammation, immune cells such as monocytes are recruited to the site of inflammation in the brain and this usually involves migration across the BBB endothelium. A key step in this process is the attachment of monocytes to the apical surface of the endothelial cells through cell surface molecules, including CD54 (intercellular adhesion molecule 1; ICAM-1) and CD106 (vascular cell adhesion molecule 1; VCAM-1) (reviewed by [4]). CD54 is the ligand for CD11/CD18 expressed by leukocytes. CD106 interacts with CD49d/CD29 on monocytes to allow the firm adhesion of monocytes onto the endothelium [4]. Both CD54 and CD106 cluster upon binding to monocytes, then migrate to the endothelial cell border to facilitate paracellular migration of the monocytes between the endothelial cells. As a consequence, the engagement and migration of monocytes across the BBB during neuroinflammation changes the barrier integrity as there must be a change in the organisation of the tight junctions and adherens junctions for paracellular migration to occur. It is the change in the expression or density of these junctional complexes, which is exploited using Electric Cell-substrate Impedance Sensing (ECIS) technology [5, 6]. Where a tight endothelial barrier exists, the conductance of electrons across it is relatively low and it therefore has a high resistance. Here there is also an inability of monocytes to move through the barrier easily. However, when a strong barrier is weakened either physically through damage or under regulation specifically to open the tight junctions, there is less resistance and thus more electrons can pass through [7–9].

Previously we have shown that human brain microvascular endothelial cells (hCMVECs) are innately programmed to respond differently to TNFα and IL-1β [10], two very important pro-inflammatory mediators produced by a range of leukocytes [11] and some resident brain cells [12, 13]. Their response profile suggested that the IL-1β activated brain endothelial cells are more primed or receptive to interaction with leukocytes such as monocytes. This was particularly evident from the chemokine secretion and expression of leukocyte adhesion molecules [10]. It was therefore the aim of this study to ascertain whether brain endothelial cells preferentially interacted with monocytes after the endothelium had been activated by IL-1β in comparison to TNFα. We hypothesised that this would be highly likely and that ECIS technology would show a greater reduction in endothelial barrier integrity under IL-1β activated conditions following monocyte addition. This was indeed observed but only with monocytes enriched using CD14^+ve^ isolation and not with untouched monocytes. Flow cytometry analysis revealed concerning issues with the purity of monocytes enriched using the untouched method (~70%), which was considerably lower than the CD14^+ve^ isolation method (>90%). These observations and other important considerations are discussed in detailed and should be considered very carefully by any researchers wishing to conduct similar studies using monocyte enriched populations.

## Materials and methods

### Cell culture

The human cerebral microvascular endothelial cell (hCMVEC) line (cat# T0259, purchased directly from ABM Good, USA) was maintained in T75 flasks coated with 1 μg/cm^2^ collagen I (Gibco) in M199 media containing 10% FBS (M199 growth media) as previously described [10].

### Immunocytochemistry

The hCMVECs were seeded into collagen I coated 96 well plates at the density of 20,000 cells per well in M199 growth media and were grown for 48 h after plating. For CD144, Zonula occludens-1 (ZO-1) and actin labelling, cells were pre-fixed with 2% paraformaldehyde (PFA) for 2 min, followed by 4% PFA fixation for 10min. The fixation solution was aspirated, and the hCMVECs were rinsed with PBS, and were permeabilized with 0.1% Triton-X in PBS (PBST) for 10 min. For CD31 immunolabelling, cells were fixed in ice cold 95% ethanol with 5% glacial acetic acid for 5 min. Cells were washed with PBS thrice before blocking with 1% BSA in PBS for 45 min. Cells were washed three times with PBST and were incubated in anti-CD31 (cat# 555444, BD Biosciences, 1:100), anti-CD144 (cat# sc6458, Santa Cruz, 1:100), anti-ZO-1 (cat# 339100, Invitrogen, 1:100) or actin (cat# R37110, Molecular Probes, 1:50) for 1 h at room temperature. Cells were washed three times with PBST and were incubated in corresponding Alexa Fluor 488 conjugated secondary antibody (Goat anti-Mouse, cat# A-11001, Invitrogen, 1:400; Goat anti-Rabbit, cat# A-11008, Invitrogen, 1:400) and nuclei stained with Hoescht 33342 (R37165, Invitrogen, 1:10,000) for 1 h at room temperature. Finally, cells were washed with PBST, and widefield image were taken on EVOS FL Auto Cell Imaging System (Invitrogen).

### FITC-dextran permeability assay

The hCMVECS were seeded into collagen I coated Transwell 0.4μm pore polycarbonate membrane inserts (cat# 3413, Costar) at the density of 20,000 cells per well in M199 growth media and were grown for 48 h. 70 kDa Fluorescein isothiocyanate (FITC)–dextran (cat# 46945, Sigma Aldrich) diluted to 10 μg/mL in M199 growth media was added to the inner well. Cells were incubated for 4 h at 37°C, and 80μL of the media from the outer well was collected for analysis. The fluorescence intensity of the collected media was measured using CLARIOstar (BMG Labtech). The fluorescence intensity of the growth media only (autofluorescence) was subtracted from the fluorescence intensity of the media collected at 4h. The fluorescence intensity of the outer well media was normalised to the blank reading (collagen coated insert with no cells).

### Western blot

The hCMVECs were seeded into collagen I coated 6 well plates at the density of 545,000 cells per well in M199 growth media and were grown for 24, 48 and 72 h after plating. Cells were lysed with lysis buffer and were heated at 90°C for 5 min. Protein was quantified using DC protein assay (cat# 5000111, Bio-Rad). 20μg of samples were separated on 4–15% precast gels (cat# 4568083, Bio-Rad) and were electroblotted to nitrocellulose. The blot was rinsed in TBST and was incubated in blocking buffer (10% non-fat milk in TBST) for 30 min with rocking. The blot was probed with a mouse monoclonal anti-occludin (cat# MAB7074, R&D systems, 1:400) or a rabbit polyclonal anti-occludin (cat# 71–1500, Invitrogen, 1:400) in immuno-buffer (2% bovine serum albumin in TBST) overnight at 4°C. The membranes were washed in TBST, and were incubated with Rabbit anti-Mouse IgG-peroxidase (cat# A9044, Sigma) or Goat anti-Rabbit IgG-peroxidase (cat# A0545, Sigma) diluted at 1:4000 in 5% non-fat milk in TBST for 2 h at room temperature. Bands on membrane were visualised by chemiluminescence (Pierce ECL Plus Western Blotting Substrate, cat# 32132, Thermo Scientific) using ChemiDoc XRS+ Systems (Bio-Rad). Membranes were stripped, washed and blocked prior to incubation with rabbit anti-GAPDH (cat# 99545, Sigma, 1:5000) at 4°C overnight. Membranes were washed and incubated with Goat anti-Rabbit IgG-peroxidase (cat# A0545, Sigma, 1:16,000) for 2 h at room temperature, then visualised by chemiluminescence as described above. The band intensity was quantified and normalized to the house keeping gene GAPDH using Image Lab Software 6.0.

### Transendothelial electrical resistance

**Fig 1:**
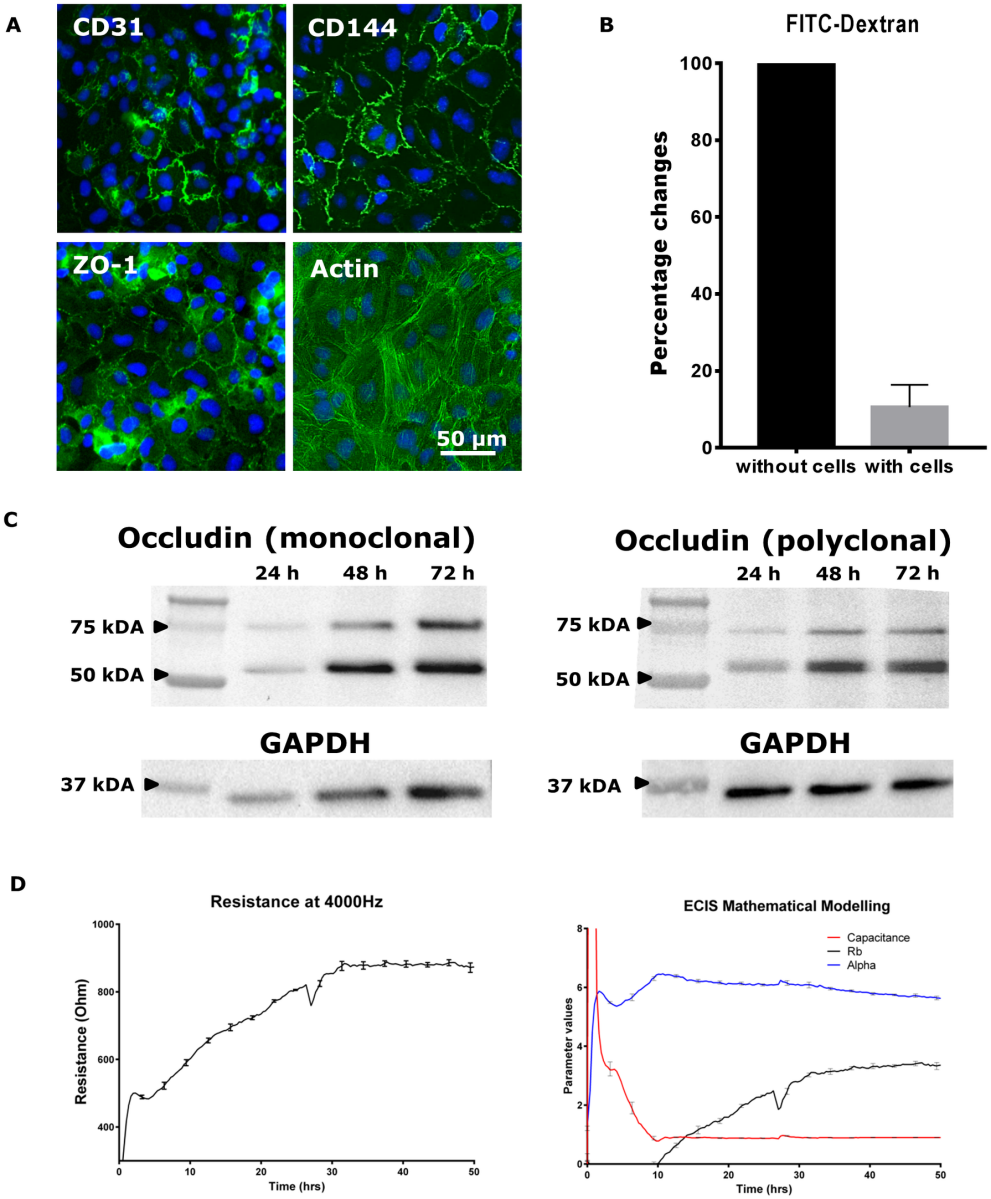
Analysis of barrier formation in endothelial monolayer. (A) Expression of endothelial junctional molecules CD31, CD144 and ZO-1 (from left to right) and actin (far right) in hCMVEC monolayer 48 h after seeding. (B) The permeability of hCMVEC monolayer to FITC-dextran 48 h after seeding (mean ± SEM, n = 4). (C) Time course analysis of endothelial tight junction molecule occludin expression at 24, 48 and 72 h post seeding of hCMVECs using Western blot using a mouse monoclonal anti-occludin (left) and rabbit polyclonal anti-occludin (right). (D) Real time measurement of electrical resistance across the endothelial monolayer as an indicator of endothelial barrier integrity. The barrier resistance (Rb) and the basolateral adhesion (Alpha) due to focal adhesions can be modelled using the ECIS Z theta software when the experiment is run using multi-frequency mode (250 to 64000 Hz). The modelling reveals that the basolateral adhesion occurs very fast and junction formation begins around 10 hours after seeding. Overall barrier resistance is the summation of both Rb and alpha, but this software function can determine whether barrier changes are predominantly due to the Rb. Data are mean ± SEM (n = 4).

Changes in endothelial barrier strength were measured using ECIS technology (Applied Biophysics) [14–16]. S1 Fig explains the principals of ECIS and measurement of barrier integrity. Fig 1D shows how the overall barrier resistance can be modelled by the ECIS software to reveal changes relating to the paracellular barrier (Rb) or to the basolateral focal adhesion (alpha). The custom ECIS plates (96W20idf) were coated with 10 mM L-cysteine, followed by coating with 1 μg/cm^2^ collagen I (Gibco). The hCMVECs were seeded at 54,000 cells per well in 100 μL of M199 growth media and were monitored until monolayer and tight junction formation has occurred, typically ~28 h post seeding [10]. For the initial experiments, multiple frequency data was collected for barrier modelling as recommended by Applied Biophysics. The alpha, Rb and capacitance were modelled using ECIS software (Applied Biophysics). Subsequently, single frequency data at 4000 Hz was collected for barrier integrity as recommended by Applied Biophysics. Once the barrier resistance reading had reached its maximum and had plateaued, indicating the formation of tight junctions, 50 μL of M199 growth media containing 1.5 ng/mL of IL-1β or 1.5 ng/mL TNFα were added to the existing 100 μL media in each corresponding well to achieve the final concentration of 0.5 ng/mL for both IL-1β and TNFα. The barrier integrity of hCMVECs was monitored for 24 h at which point enriched monocytes were added to respective wells. Enriched monocytes were counted and added at either 54,000 monocytes (1:1 effector to target ratio) or 5,400 monocytes (1:10 effector to target ratio) to corresponding wells. No monocyte controls received media only. Endothelial barrier resistance was monitored for the next 30 h. The results are represented as the average Resistance ± SEM of 3 replicates.

### Blood collection and monocyte isolation from PBMCs

Blood samples were obtained from healthy donors following informed consent, where ethical approval was granted by the University of Auckland Human Ethics Board (ethics number **014035)**. All donors provided written approval for the use of their blood in this study. Blood was collected by venepuncture into sterile EDTA coated blood collection tubes (Becton Dickinson). Peripheral blood mononuclear cells (PBMCs) were isolated immediately following venipuncture using Ficoll-Hypaque (Sigma Aldrich) density gradient centrifugation with Leucosep tubes (Greiner Bio-One) as per the manufacturer’s instructions.

Monocytes were isolated from PBMC using two different methods in parallel. Half of the PBMCs were used for positive selection by CD14 MicroBeads (cat#130-010-201, Miltenyi Biotec), whereas the other half of the PBMCs were used for untouched selection using the pan monocyte isolation kit (cat# 130-096-537, Miltenyi Biotec). Both isolation methods were performed with manual columns strictly according to the manufacturer’s protocol. The purity of the monocytes was evaluated using fluorescence staining of the fluorochrome conjugated CD14-PE antibody (cat# 301806, Biolegend) and CD16-APC antibody (cat# 302012, Biolegend). The stained cells were acquired using Accuri C6 flow cytometer (BD Bioscience) and were analysed using FlowJo version 7.6.5. Cell debris and dead cells were excluded from the analysis based on scatter signals and 7AAD (cat# 420404, Biolegend) signal.

### Flow cytometric analysis of monocyte populations

Debris and platelets were excluded from the live gate based on the forward scatter/side scatter. In BD instruments (i.e. Accuri C6), cell doublets and clumps can be discriminated using area scaling, based on the disproportions of area and height forward scatter and side scatter hierarchically. The dead cells from singlet population were discriminated using 7AAD (cat# 420404, Biolegend) stain and were excluded from the gate. The 7AAD negative single cells were plotted as FSC-A vs SSC-A plot. Monocytes were identified by their position based on the forward and side scatters. The monocyte subsets (CD14^hi^ CD16^low^, CD14^hi^CD16^hi^, and CD14^dim^CD16^hi^) were distinguished by plotting CD14 (LPS co-receptor) signal against CD16 (FcγIIIR) signal.

### Flow cytometry of hCMVECs

Cell surface expression of leukocyte adhesion molecules was measured using flow cytometry. In brief, cells were seeded in 1 μg/cm^2^ collagen I (Gibco) coated T25 flasks and were grown to confluence in M199 growth media. Once cells reached 100% confluency, media was replaced with fresh M199 growth media only, or M199 growth media containing either 0.5 ng/mL IL-1β or 0.5 ng/mL TNFα. Cells were treated for 37°C for 24 h, and were detached using EDTA-based Versene (Gibco). The cell suspension was adjusted to final cell concentration of 1x10^6^ cells per mL in cold FACS buffer (1% FBS in PBS). Fluorochrome-conjugated antibodies anti-CD54-FITC (cat#353108, Biolegend) and anti-CD106-PE (cat# 305806, Biolegend) were added to cells at previously optimised titrations [10]. Cells were incubated for 10 min on ice, and were washed twice in 1 mL of cold FACS buffer followed by centrifugation at 400x g for 10 min. The cell pellet was re-suspended in 100μl of FACS buffer. The stained cells were acquired using an Accuri C6 flow cytometer (BD Bioscience). Gating strategy was done as previously described in [10], where 7AAD was used to stained dead cells and define the live-cell gate.

### Cytokine measurements

The hCMVECs were seeded into collagen I coated 24 well plates at the density of 80,000 cells per well. Once cells reached confluence they were treated for 24 h with M199 10% growth media only (control/vehicle), or 0.5 ng/mL of IL-1β or 0.5 ng/mL TNFα in growth media. Then 100 μL of conditioned media was collected and centrifuged at 400x g for 5 min at 4°C to remove debris. Samples were stored at -20°C until required. The concentrations of soluble CD54 (cat#560269, BD Bioscience), soluble CD106 (cat#560427, BD Bioscience) and MCP-1 (cat#558287, BD Bioscience) in conditioned media were quantified using multiplexed cytometric bead array (CBA, BD Biosciences). Samples were analysed using BD Accuri C6 flow cytometer (BD Bioscience). The mean fluorescence intensity values were converted into cytokine concentrations based on the 10-point standard curve (0 to 5000 pg/mL) using FCAP Array software (BD version 3.1).

### Statistics

Data are described in the text and presented graphically as mean ± SEM for data from one representative experiment. Experiment was repeated at least 3 times. Statistical analyses were performed using GraphPad Prism^®^ 6.07 (GraphPad Software Inc, LaJolla, CA). Data were analysed using Student’s t-test. Graphical representations of p values are as follows: *, p ≤0.05; **, p ≤0.01; ns, p >0.05.

## Result

### Pro-inflammatory activation of hCMVECs

The brain endothelial hCMVEC monolayer exhibits a cobblestone phenotype and expresses junctional molecules including CD31, CD144, ZO-1 and occludin (Fig 1). The protein expression of tight junction molecule occludin is detectable at 24 h post-seeding, and the level of occludin expression at 48 h and 72 h post-seeding is approximately 2.3 times higher than at 24 h (Fig 1C). The hCMVECs monolayer formed a barrier at 48 h, which reduced the permeability of FITC-dextran across the Transwell inserts by approximately 90% (Fig 1B). Previous study also demonstrated that the hCMVECs were able to form a barrier with transendothelial electrical resistance (TEER) of ~60 Ω cm^2^ measured using EVOM2 Voltohmmeter and STX3 electrode [17]. The barrier resistance of hCMVEC monolayer was measured using the real-time and label-free electric cell-substrate impedance sensing (ECIS) Zθ technology in multi-frequency mode. The mathematical modelling of the multi-frequency data using the ECIS Z theta software reveals that the endothelial monolayer form its basolateral adhesion (Alpha; blue curve) very quickly whereas junction formation (barrier resistance, Rb) begins around 10 hours after seeding (Fig 1D). The overall resistance at 4000 Hz is the summation of the Alpha, Rb and capacitance (Cm), but it can be used to monitor real-time changes in endothelial barrier function (Rb) as suggested in the literature [7] and shown here in Fig 1D. The basolateral adhesion Alpha and membrane capacitance plateau at 20–40 hours (Fig 1D), therefore, the overall resistance acquired at 4000 Hz is used as the **real time** surrogate of when the barrier has formed, which is typically between 20–30 hours post seeding.

**Fig 2:**
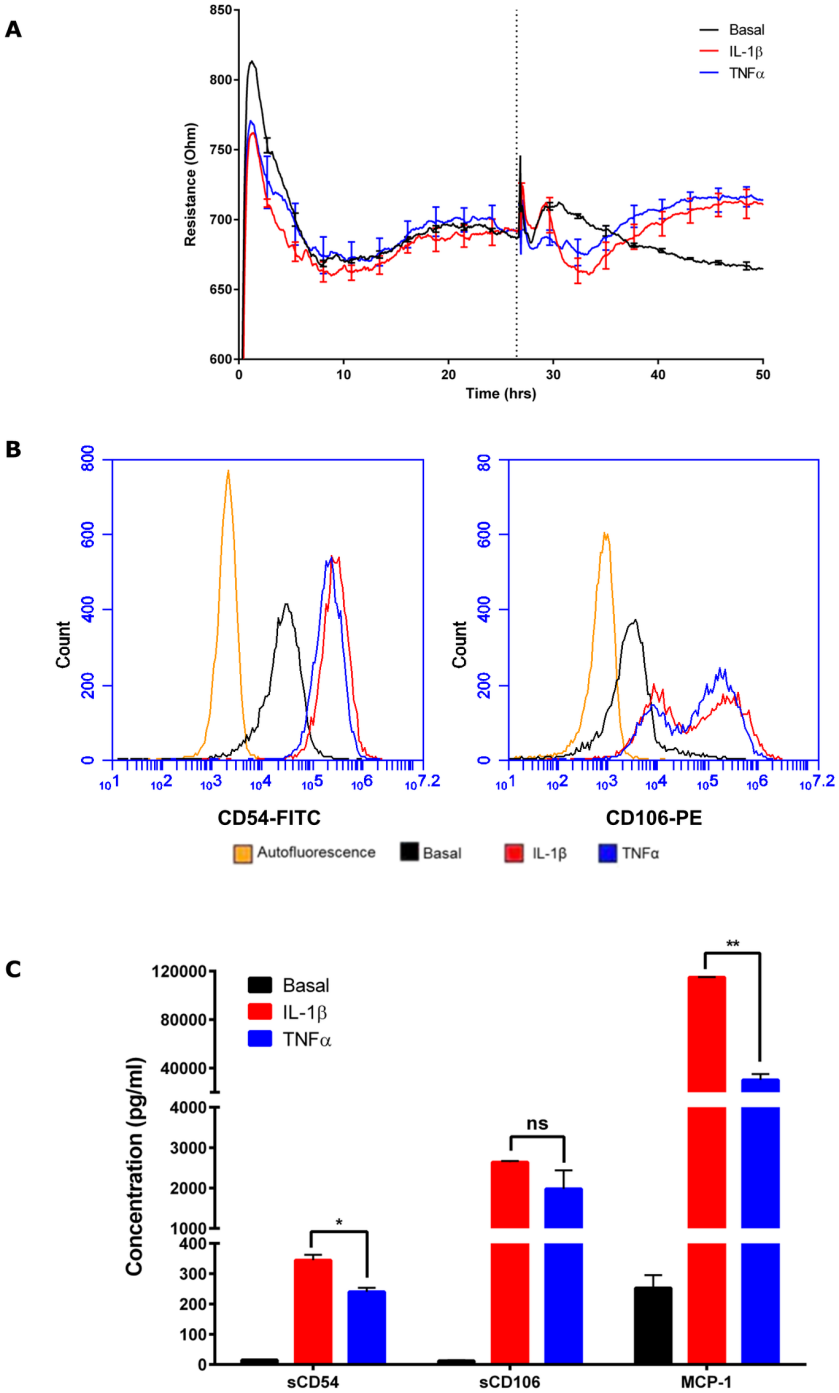
Analysis of endothelial activation following treatment with IL-1β and TNFα stimulation for 24 h. (A) Real time measurement of electrical resistance across endothelial monolayer as an indicator of endothelial barrier integrity. Media containing IL-1β (red), TNFα (blue) or vehicle only (black) are added when endothelial cells have formed tight barriers approximately 20 h after seeding, indicated by the dotted vertical line. Data are mean ± SEM (n = 4) (B) The flow histograms show the cell surface expression levels of leukocyte adhesion molecules CD54 (ICAM-1) and CD106 (VCAM-1) under basal condition (black), and 24 h post-stimulation with 0.5 ng/mL IL-1β (red) or 0.5 ng/mL TNFα treatment (blue). Orange line represents the autofluorescence of the cells. (C) Secretion of soluble CD54 (sCD54), soluble CD106 (sCD106) and MCP-1 following activation by IL-1β (0.5 ng/mL) and TNFα (0.5 ng/mL) for 24 h. The concentration of cytokines was measured in conditioned media using multiplex CBA. Data show the mean ± SEM (n = 3). Data were analysed using Student’s t-test, * represents p ≤0.05; ** represents p ≤0.01; ns represents p >0.05.

Treatments (control media, IL-1β and TNFα treatments) were added when the barrier had formed and the endothelial barrier integrity was then measured continuously after addition of the pro-inflammatory cytokines to capture the temporal changes in barrier resistance (Fig 2). There is an acute increased in basal barrier resistance during the first 4–8 h following addition of fresh media, after which the barrier resistance readings gradually returned to the initial readings (~700 Ω). Both IL-1β and TNFα (0.5 ng/mL) altered the barrier integrity immediately but with subtle differences. IL-1β stimulation caused a transient increase in barrier resistance within a couple of hours, followed by a reduction in barrier resistance below basal, which lasted for 10–12 h. The endothelial response to TNFα stimulation lacked the initial transient increase in barrier resistance and remained lower than the basal level for 10–12 h. After this initial period of depression, the barrier resistance increased for ~30–40 h for both TNFα and the IL-1β response (Fig 2A).

Leukocyte recruitment is an important step preceding transendothelial migration and involves the capture, rolling, and activation of the leukocyte on the apical face of endothelial cells [18]. We have shown previously that the hCMVECs express low levels of CD54 and CD106 basally and that expression of these key attachment molecules peaks around 24 h after activation of the hCMVECs [10]. Fig 2B confirms the high level CD54 and CD106 expression induced by IL-1β and TNFα (0.5 ng/mL) at 24 h, which is the time point of monocyte addition used in this study (Fig 2B, S1 Data). Generally, IL-1β increases CD54 and CD106 expression to a greater extent in comparison to TNFα [10]. In addition, IL-1β mediates a greater level of cytokine secretion from hCMVECs in comparison to TNFα, including the potent monocyte chemoattractant, MCP-1, soluble CD54 and soluble CD106 (see Fig 2C and shown extensively in [10]). Collectively, this suggests that monocytes maybe preferentially recruited under the IL-1β inflammatory milieu in comparison to that occurring with TNFα. This was therefore assessed using ECIS technology.

### Comparison of monocyte-induced endothelial barrier disruption following TNFα or IL-1β activation using ECIS technology

**Fig 3:**
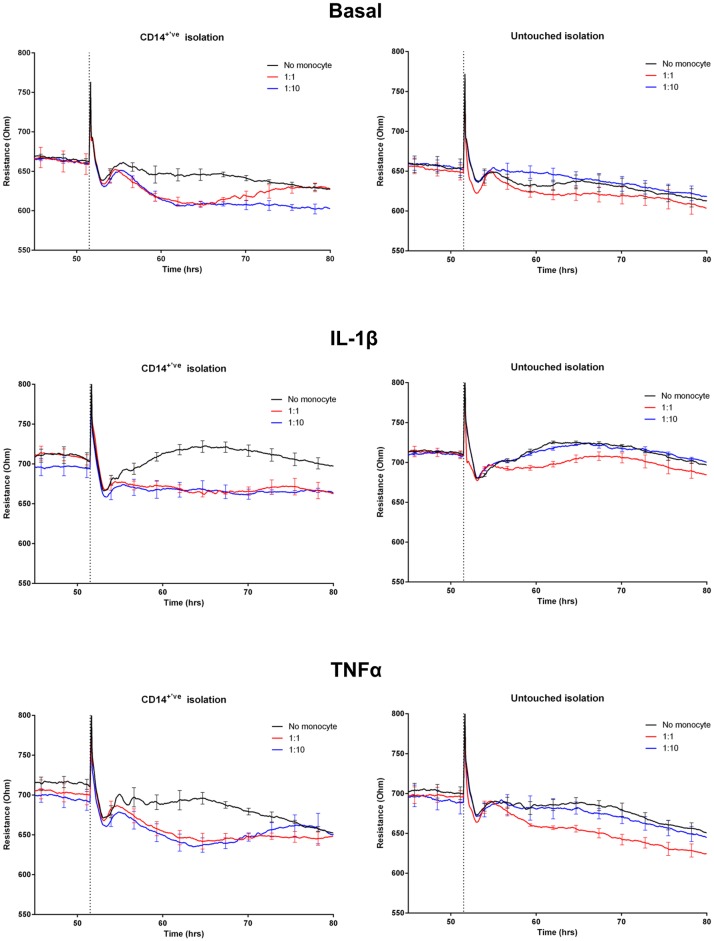
Representative temporal profile of endothelial barrier integrity following the addition of enriched monocytes, reflecting monocyte-endothelial activation. The endothelial barrier resistance was measured using ECIS Zθ technology. The top panels are under basal conditions, middle panels are IL-1β activated and bottom panels are TNFα activated endothelium, which were activated 24 h prior to monocyte addition. Monocyte addition is demarcated by the dotted vertical line (~52 h). Monocytes isolated using CD14^+ve^ method and the untouched method were added onto the endothelial cells at the effector to target ratio of either 1:1 (red line) or 1:10 (blue line). Black line represents endothelial barrier integrity of cells that received media only (no monocytes). Each line represents the mean ± SEM of 4 wells.

**Fig 4:**
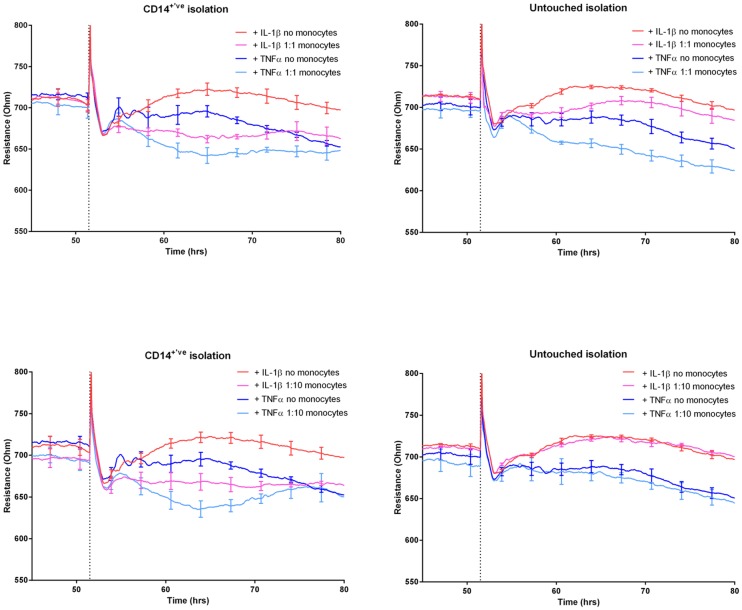
Monocytes isolated by CD14^+ve^ isolation mediate a greater degree of barrier disruption than untouched monocytes. Data are segregated based on monocyte isolation and ET ratio (monocytes to endothelial cells). Top panels show effects at 1:1 ratio with 1:10 ratio for the bottom panels. The monocytes isolated by CD14^+ve^ enrichment are used in the left panels and the untouched monocytes in the right panels. The red and pink curves represent the IL-1β treated endothelium, where the red curve does not have monocytes added. The blue curves represent the TNFα treated endothelium, where the dark blue has not had monocytes added (see legend in figure). Each line represents the mean of 4 wells (±SEM).

The primary aim of this study was to assess whether monocyte-mediated barrier disruption was different following IL-1β activation in comparison to TNFα. We hypothesised that due to the propensity of the endothelial cells to respond more potently to IL-1β [10], that there would be a pronounced difference, where IL-1β would cause a greater extent of monocyte attachment and subsequent migration across the endothelium and thus a greater change in the barrier integrity. However, the changes in barrier integrity following monocyte addition between the IL-1β and TNFα activation were more subtle than hypothesised (see Figs 3 and 4, S2 Data) and appeared to be influenced greatly by the method of monocyte enrichment.

### CD14 positive isolated monocytes mediate greater loss of BBB integrity than untouched monocytes

Monocytes enriched using CD14^+ve^ isolation caused a greater reduction in barrier resistance than monocytes enriched using the untouched method (Figs 3 and 4). This is most evident if you compare the effect at the 1:10 ratio (which is 1 monocyte to 10 endothelial cells) for the CD14^+ve^ isolation to the 1:1 untouched ratio under any of the conditions (Figs 3 and 4). There is clearly a greater effect at 1:10 ratio for the CD14^+ve^ monocytes, even though the 1:1 untouched ratio should have 10 times as many monocytes. It is also very clear that the 1:10 ratio of untouched monocytes has no effect in any of the conditions tested. In terms of a difference between the IL-1β and TNFα activated endothelium, there is a temporal difference following CD14^+ve^ monocyte addition. Wherein the reduction in barrier resistance mediated by the monocytes lasts longer in the IL-1β activated endothelium, whereas under the TNFα activation the barrier resistance has returned to control faster (Figs 3 and 4). Here we would conclude a greater extent of endothelial barrier disruption with monocytes enriched by CD14^+ve^ isolation on endothelium activated with IL-1β in comparison to TNFα. As the effects of the untouched monocytes on the endothelial cells were considerably less overall, the differences mediated through IL-1β and TNFα are therefore less obvious.

It is important to note that the CD14^+ve^ isolation and untouched isolation were conducted in parallel for every donor. Therefore, the data shown in Figs 3 and 4 is from the same donor with all experiments conducted simultaneously. These data represent isolation-dependent effects observed with at least 5 different donors.

### Comparison of the purity of enriched monocyte isolated using two different methods

**Fig 5:**
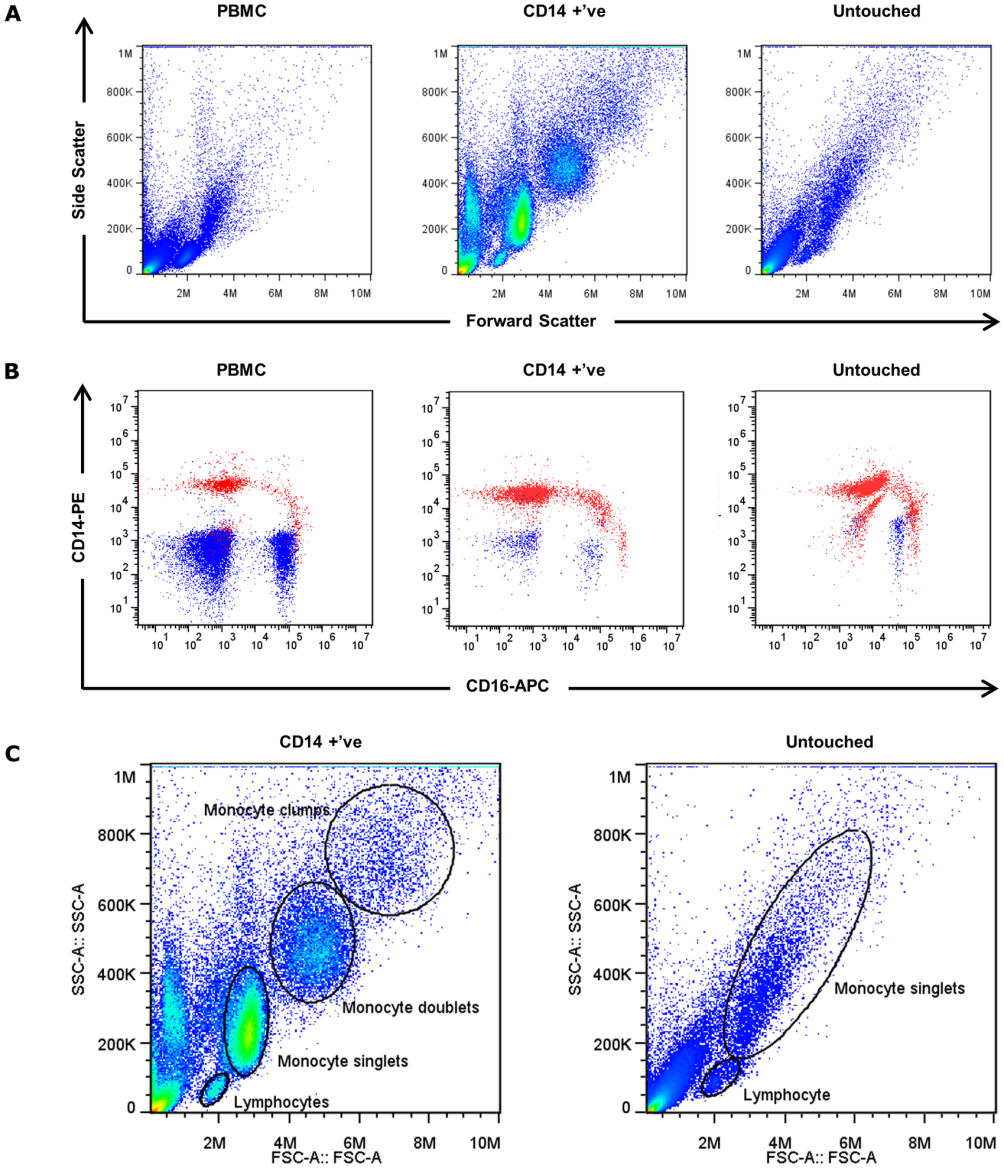
Analysis of enriched monocyte purity comparing the two monocyte isolation methods conducted in parallel. (A) Representative forward and side scatter flow diagrams (n = 5) of PBMC (left), CD14 positively isolated monocyte (centre) and untouched monocyte (right) from the same donor. (B) Comparison of monocyte subsets (red) and lymphocyte (blue) in PBMCs and in enriched monocytes, identified using PE-conjugated anti-CD14 antibody and APC-conjugated anti-CD16 antibody. (C) Monocyte and lymphocyte gating strategy highlighting the monocyte singlet gates and doublets to emphasise the difference between the two methods. More extensive gating showing the hierarchical strategy for defining the singlet gates is shown in S4 Fig.

In parallel to the ECIS experiments, flow cytometry was conducted on all of the donor PBMC samples and the monocyte enriched fractions. This data was then scrutinised to assess whether there were any differences in monocyte yield or purity that could explain the substantial differences observed between the two isolation methods (Fig 5, S3 Data).

Fig 5A shows the scatter plots of the initial PBMC sample and for the subsequent monocyte isolations. The CD14^+ve^ isolation yields highly enriched monocyte populations with most of the cells present in the monocyte singlet gate (see also S3 Fig). Of particular note however, is that there are additional clusters (larger FSC/SSC) of monocytes that can be defined as doublets and multiples of cells based on single cell discrimination (see methods and S3 Fig). On average the purity of the monocytes in the CD14^+ve^ enriched fraction was always greater than 90% and on average 94% pure. This is based on the number of cells in the singlet gate only and does not include the doublets, which may underestimate the purity. In contrast to this, the purity of the monocytes produced in the untouched method was rarely above 90% and on average was only ~70% (see Table 1). There was less evidence for the monocytes clumping or forming doublets consistent with an untouched approach, contrary to the CD14^+ve^ isolation (Fig 5 and S3 Fig).

**Table 1:** Comparison of purity of monocytes enriched using the two isolation methods. Mean ± SD of 5 independent experiments.

	Monocyte Purity (%)
**PBMC**	14.1 ± 2.78
**CD14^+ve^ isolation**	93.36 ± 1.02
**Untouched isolation**	70.30 ± 11.26

We also compared the monocyte subsets (CD14 vs CD16) enriched using the two methods (Fig 5B and S3 Fig). The CD14^+ve^ isolation method yielded the CD14^hi^/CD16^low^ monocyte (classical monocyte), and the CD14^hi^/CD16^hi^ monocyte (intermediate monocyte), but not the CD14^low^ /CD16^hi^ monocytes (non-classical monocyte), as anticipated. The untouched method on the other hand enriched all three classes of the monocyte subsets, but the CD14 signal intensity also appeared to be shifted. However, in some instances CD16^hi^ granulocytes were also present in the enriched fraction and these could be back gated to the granulocyte position on the FSC/SSC plots (see S7 Fig).

There are indeed huge differences in the forward and side scatter plots when comparing the CD14^+ve^ isolation method and the untouched. In addition, in several of the untouched isolations the monocyte purity was very low (<50%), meaning the majority of the enriched fraction was non-monocytic. The untouched isolations produced highly inconsistent purities of enriched monocytes (Table 1), even though it was conducted in parallel (at exactly the same time) as the CD14^+ve^ isolation method (see S1–S7 Figs). This demonstrates a number of important differences between the two isolation methods, which may have implications for downstream experiments and contribute to the effector function of the monocytes on the endothelial barrier integrity

## Discussion

The breakdown of the BBB is a feature of neuroinflammation occurring in a range of neurological conditions (reviewed by [19]). The key cellular component of the BBB is the endothelium, which expresses high levels of tight junction and adherens junction. These molecules provide a paracellular barrier to the movement of immune cells from the blood into the brain. Immune cell trafficking into the central nervous system is very low under normal physiological conditions and this confers the brain’s immune specialised status. However, this dynamic changes greatly during neuro-inflammation. The BBB endothelium can be activated resulting in the innate ability to secrete a variety of inflammatory mediators and to up-regulate expression of adhesion molecules capable of recruiting a range of blood borne leukocytes [20]. We have previously shown that human brain endothelial cells (hCMVECs) respond differentially to the pro-inflammatory cytokines IL-1β and TNFα [10]. In this regard, IL-1β induced higher expression of key leukocyte adhesion molecules CD54 and CD106. IL-1β was more potent than TNFα at inducing secretion of IL6, IP10, MCP-1, GCSF and GMCSF. Whereas TNFα induced a greater level of IL-8 and RANTES secretion. Taken together this suggests differential regulation of the brain vascular endothelial cells under these inflammatory conditions. These observations prompted us to investigate whether endothelial monocyte migration and barrier integrity is also differentially regulated by these two cytokines. ECIS technology currently represents the gold standard for monitoring of endothelial barrier integrity due to its autonomous real time capacity, which therefore can measure both acute and chronic changes in barrier functions [7]. This study would simply not be possible without the real-time nature of the ECIS recordings and capacity to measure 96 wells simultaneously.

Under basal conditions, the hCMVECs form a strong barrier and express very low levels of inflammatory cytokines and adhesion molecules. Following activation with IL-1β and TNFα there is an acute weakening of the barrier integrity, prior to a pronounced increase in barrier strength which coincides with increased expression of CD54 and CD106. We considered that this increase in barrier strength was the endothelial cells’ innate response in preparation for leukocyte attachment and presumptive paracellular migration. We hypothesised that IL-1β would promote a greater degree of monocyte migration and consequent change in barrier integrity, which would be consistent with the greater expression of inflammatory chemokines and adhesion molecules [10]. This was indeed observed but was really only evident where the monocytes were isolated using the CD14^+ve^ isolation method. It can be observed in the data shown in Figs 3 and 4, that the reduction in barrier integrity is decreased by the monocytes for longer during the IL-1β activated state, in comparison to the TNFα activation of the brain endothelial cells. In the IL-1β activated endothelium, the monocytes reduce the barrier for at least 24 h longer than TNFα and at this stage we do not know the full mechanism of action but this will be the focus of a future study.

In this study monocytes were isolated using two different isolation methods and for all of the donors the isolations were conducted in parallel with all subsequent analysis conducted simultaneously. Monocytes were isolated by the CD14^+ve^ isolation method and the so called untouched pan isolation, both kits from Miltenyi Biotec (full details in methods). In principle, the CD14^+ve^ isolation uses the classical monocyte marker CD14 to pull out monocytes (positive isolation). However, not all monocytes express high levels of CD14 [21], with the non-classical CD16^hi^/CD14^dim^ subset not being represented [21]. The untouched monocyte method has the advantage that it should enrich for all monocyte subsets and works by negative selection, hence untouched. However, this particular kit from Miltenyi Biotec does not declare which antibodies are included in the antibody cocktail, which should be a matter of concern when using this kit. We originally used both as it was suggested that the non-classical monocytes could give a different response even though they are of relatively low abundance. It became very clear from the very first donor and then every subsequent donor that the method of monocyte enrichment had a massive effect on the capacity of the resultant monocytes to affect barrier integrity. This point is most obviously demonstrated by comparing the 1:1 ratio of untouched monocytes to the 1:10 ratio of CD14^+ve^ isolated monocytes (Fig 3) under any of the conditions (basal or activated endothelium). Note that 1:10 ratio is 1 monocyte (effector) to 10 endothelial cells (target) so this is effectively a very low ratio and lower than that used in most studies [22–24]. Next we diligently assessed the purity of the monocyte enriched fractions by flow cytometry, which was conducted in parallel with the same enriched cells being added onto the ECIS system. The flow-cytometry analysis revealed distinct and concerning differences between the monocyte isolation methods above that expected from their principle design. Of considerable concern was that the untouched monocyte enrichment was highly variable (consistently below 80% monocyte purity). Contrary to this, the CD14^+ve^ isolation produced a highly pure monocyte yield, which was consistently above 90%. In addition, we noted that the CD14^+ve^ isolation promoted a greater degree of doublets and clumping, presumably due to binding or crosslinking multiple cells with the isolation beads. Although the purity of the untouched yield was concerning, the difference in monocyte number alone does not account for the effects observed on ECIS, which suggests that the isolation method directly influences the subsequent behaviour of the monocytes. One possible explanation is that direct conjugation of CD14 by the beads results in direct activation of the monocytes, which then influences their behaviour analogous to activation by a danger signal. We did not measure activation markers expressed by the enriched monocytes in this study partly due to not having enough monocytes to do this and also the importance of this analysis was not realised until after the fact. Another possible explanation for the difference may be due to the non-monocytic cells present in the untouched enriched monocytes. These non-monocytes are mostly T cells with a few NK cells in some harvests (S4–S7 Figs). It is possible that these lymphocytes could directly influence the behaviour of the monocytes or the endothelial cells. The presence of allogeneic NK cells could be problematic and cause NK-mediated killing through MHC mismatch, which is also one of the reason these studies are done with enriched cells rather than directly with PBMC. The important point we want to make here is that the presence of these non-monocytic cells is often ignored and in a number of studies the purity of the monocyte harvest is not even mentioned [16].

We conclude that the purity of the isolated monocytes is imperative, especially when assessing the role of monocytes in functional studies. Isolation of pure populations of monocytes from PBMCs allows us to differentiate the effect of monocyte from other immune cells such as granulocytes, and lymphocytes, which could potentially disrupt endothelial barrier integrity too. Other studies have also implicated the importance of selecting the appropriate isolation methodology. The isolation method and the monocyte purity could affect the phagocytic capacity, phenotype of monocyte and their cytokine production due to the high plasticity of monocytes [25, 26]. In this study, we ended up comparing monocyte isolation methods, not by design but by necessity due to the differing results they produced. We therefore highlight the conundrum for researchers to contemplate and consider, which resolves around yield, purity, consistency, type of monocytes harvested, and potential activation status of the cells. All of this needs to be resolved before even considering the endothelial paradigm.

ECIS is undoubtedly very powerful due to the temporal nature and continuous measurement of barrier function [14, 27]. The complexity and intricacy of this study simply would not be possible to conduct using other technology such as the hand held EVOM voltmeter (first-hand experience). Due to the temporal monitoring of barrier integrity long term, ECIS has shown that there is a greater degree of compromise or loss of integrity in IL-1β activated endothelium following monocyte addition. Whether this translates to a greater extent of monocyte migration across the endothelial barrier will be investigated in a future study.

## Supporting information

S1 Fig Barrier resistance measured using ECIS technology. Schematic shows the principle of ECIS (electric cell impedance sensing) and how changes in endothelial barrier resistance/integrity following monocyte addition are measured. The overall barrier integrity is a combination of both the basolateral adhesion strength (alpha) and also the paracellular barrier strength (Rb). The large purple cell represents a monocyte attaching to a strong endothelial barrier, where the resistance is high and little current is allowed to pass (blue dotted lines). In the lower panel, the monocyte has begun to migrate though the paracellular space between two opposing endothelial cells and dissociated the tight junction complexes, which allows more current to flow through the paracellular space (red arrows). It is this conductivity which is measured by ECIS and gives the measure of barrier resistance or barrier integrity. In the simplest of terms, when the barrier opens there will be a reduction in resistance and vice versa. Therefore, greater monocyte migration equates to greater reduction in resistance. ECIS measures this in a temporal (real-time) autonomous manner [7–9].
(TIF)

S2 Fig Modelling of monocyte mediated barrier disruption using ECIS. As explained in SF1 and shown in Fig 1D, the ECIS modelling software can indicate which component of the barrier is being affected by the treatment. Here the changes in Rb are very similar to the overall changes in the barrier resistance for both the untouched and CD14 positively isolated monocytes, which were added onto the barrier following activation by IL-1β.
(TIF)

S3 Fig The gating strategy hierarchy to define 7AAD negative singlet cell populations and the differentiation of monocytes and lymphocytes from a heterogeneous cell population. The top row represents the gating strategy for PBMC, middle row for CD14^+ve^ isolation and bottom for untouched isolation. First column (left) represents all of the events acquired, and the live cell gate shows the exclusion of debris by gating around the live cells based on cell size and density. Cell doublets and cell clumps from the live cell gate are excluded using area scaling based on the disproportions of area and height forward scatter (second column) and side scatter (third column) hierarchically. Column four shows the exclusion of 7AAD positive dead cells from the singlet population. The 7AAD negative singlet populations are plotted as FSC vs SSC to allow the differentiation of subpopulations of monocyte and lymphocyte (column five). Column six (far right) shows the subsets of monocyte population (red) and lymphocyte (blue), identified using PE-conjugated anti-CD14 antibody and APC-conjugated anti-CD16 antibody.
(TIF)

S4 Fig Data for donor 2. Data shows single cell data dot plots for SSC/FSC revealing the monocyte and lymphocyte gates. These were then compared for CD14 and CD16 expression to show the different monocyte subsets (red) and lymphocytes (blue) present. Note the particularly low yield of monocytes at only 38% in the untouched harvest. The CD16 high (blue cells) are NK cells present in the lymphocyte gate.
(TIF)

S5 Fig Data for donor 3. Data shows single cell data dot plots for SSC/FSC revealing the monocyte and lymphocyte gates. These were then compared for CD14 and CD16 expression to show the different monocyte subsets (red) and lymphocytes (blue) present. Note the low yield of monocytes at only 40.8% in the untouched harvest. The CD16 high (blue cells) are NK cells present in the lymphocyte gate.
(TIF)

S6 Fig Data for donor 4. Data shows single cell data dot plots for SSC/FSC revealing the monocyte and lymphocyte gates. These were then compared for CD14 and CD16 expression to show the different monocyte subsets (red) and lymphocytes (blue) present. This was the only donor to produce an untouched monocyte yield above 90%. Note the lack of NK cells post isolation for this donor.
(TIF)

S7 Fig Data for donor 5. Data shows single cell data dot plots for SSC/FSC revealing the monocyte, lymphocyte and presence of a granulocyte gate. These were then compared for CD14 and CD16 expression to show the different monocyte subsets (red), lymphocytes (blue) and granulocytes (green). The CD16^hi^ (blue cells) are NK cells present in the lymphocyte gate and the CD16^hi^ (green cells) are neutrophils. The presence of the neutrophils was of concern in the untouched fraction, however, this could not be predicted as the antibody cocktail for the untouched assay is not made available to users.
(TIF)

S1 Data Original FCS files for Fig 2B. (ZIP)

S2 Data Original abp file for Figs 3 and 4. Requires ECIS software to access the data. Software is available for downloading from Applied Biophysics (http://www.biophysics.com/download.php).
(ABP)

S3 Data Original FCS file for Figs 3 and 4. The data file for S3 Data is located at Figshare’s online digital repository at https://figshare.com/s/8fd60608b49e782de618.
(DOCX)

## Abbreviations

BBB: blood brain barrier
ECIS: Electric Cell-substrate Impedance Sensing
FSC: forward scatter
hCMVECs: human cerebro-microvascular endothelial cells
IL-1β: interleukin-1β
MCP-1: monocyte chemoattractant protein
PBMC: peripheral blood mononuclear cells
SSC: side scatter
TNFα: tumour necrosis factor alpha
