# Supplementary figures and images for: ECIS technology reveals that monocytes isolated by CD14+ve selection mediate greater loss of BBB integrity than untouched monocytes, which occurs to a greater extent with IL-1β activated endothelium in comparison to TNFα
Source: PLoS One. 2017 Jul 21;12(7):e0180267. doi: 10.1371/journal.pone.0180267 (PMC5521748; doi:10.1371/journal.pone.0180267)

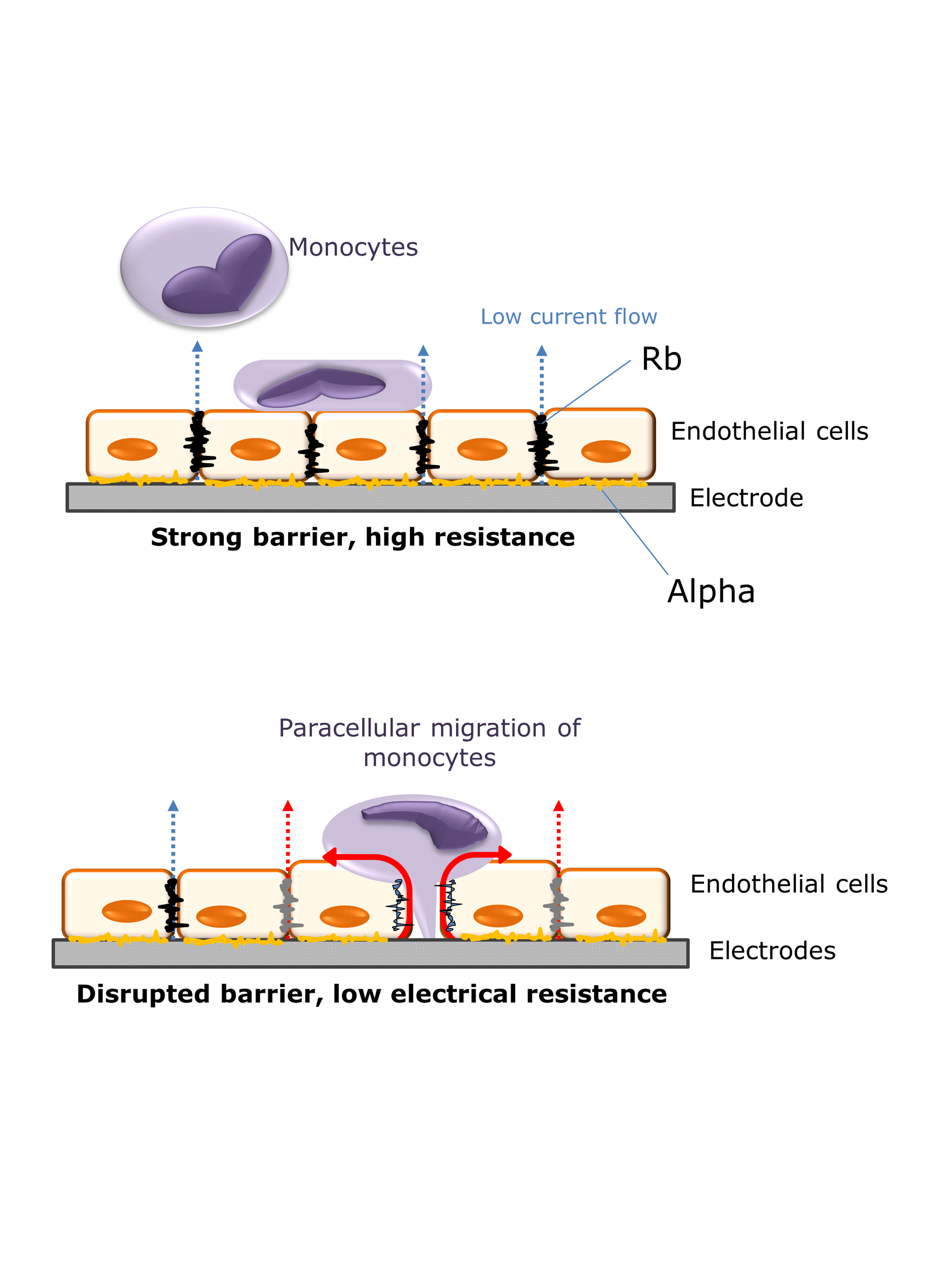

Supplement: S1 Fig — Schematic shows the principle of ECIS (electric cell impedance sensing) and how changes in endothelial barrier resistance/integrity following monocyte addition are measured. The overall barrier integrity is a combination of both the basolateral adhesion strength (alpha) and also the paracellular barrier strength (Rb). The large purple cell represents a monocyte attaching to a strong endothelial barrier, where the resistance is high and little current is allowed to pass (blue dotted lines). In the lower panel, the monocyte has begun to migrate though the paracellular space between two opposing endothelial cells and dissociated the tight junction complexes, which allows more current to flow through the paracellular space (red arrows). It is this conductivity which is measured by ECIS and gives the measure of barrier resistance or barrier integrity. In the simplest of terms, when the barrier opens there will be a reduction in resistance and vice versa. Therefore, greater monocyte migration equates to greater reduction in resistance. ECIS measures this in a temporal (real-time) autonomous manner [7–9]. (TIF) [file pone.0180267.s001.tif]

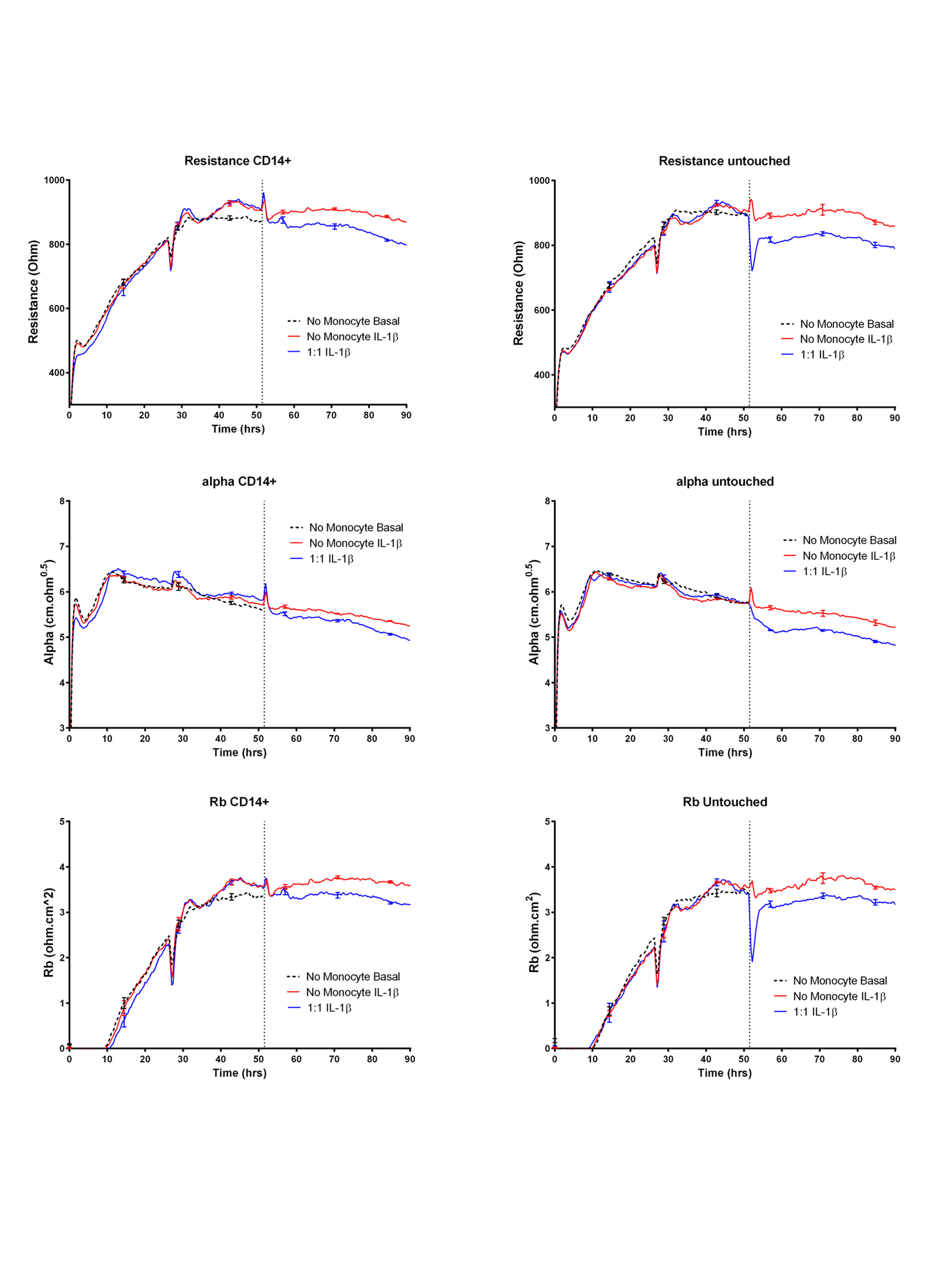

Supplement: S2 Fig — As explained in SF1 and shown in Fig 1D, the ECIS modelling software can indicate which component of the barrier is being affected by the treatment. Here the changes in Rb are very similar to the overall changes in the barrier resistance for both the untouched and CD14 positively isolated monocytes, which were added onto the barrier following activation by IL-1β. (TIF) [file pone.0180267.s002.tif]

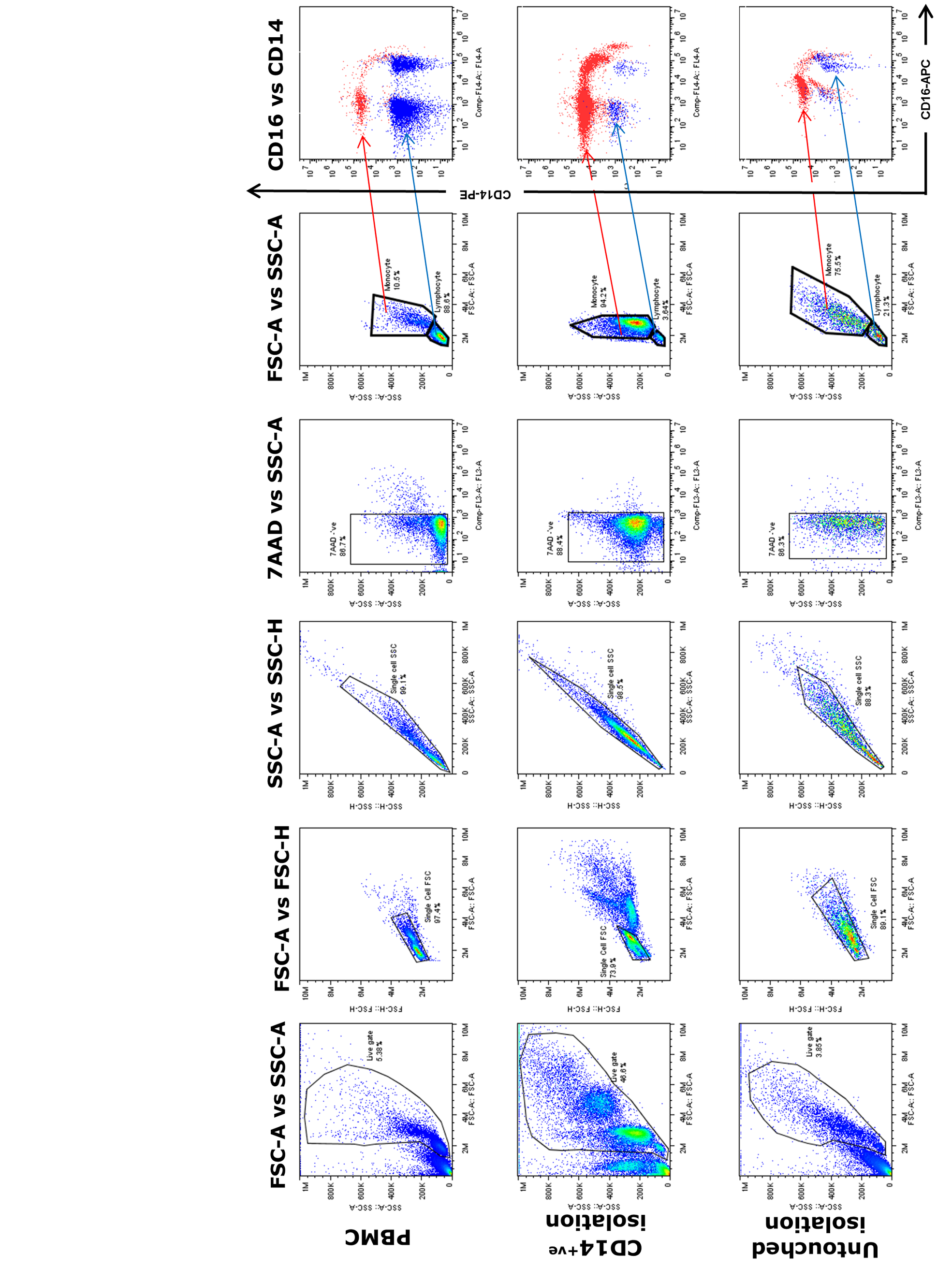

Supplement: S3 Fig — The top row represents the gating strategy for PBMC, middle row for CD14+ve isolation and bottom for untouched isolation. First column (left) represents all of the events acquired, and the live cell gate shows the exclusion of debris by gating around the live cells based on cell size and density. Cell doublets and cell clumps from the live cell gate are excluded using area scaling based on the disproportions of area and height forward scatter (second column) and side scatter (third column) hierarchically. Column four shows the exclusion of 7AAD positive dead cells from the singlet population. The 7AAD negative singlet populations are plotted as FSC vs SSC to allow the differentiation of subpopulations of monocyte and lymphocyte (column five). Column six (far right) shows the subsets of monocyte population (red) and lymphocyte (blue), identified using PE-conjugated anti-CD14 antibody and APC-conjugated anti-CD16 antibody. (TIF) [file pone.0180267.s003.tif]

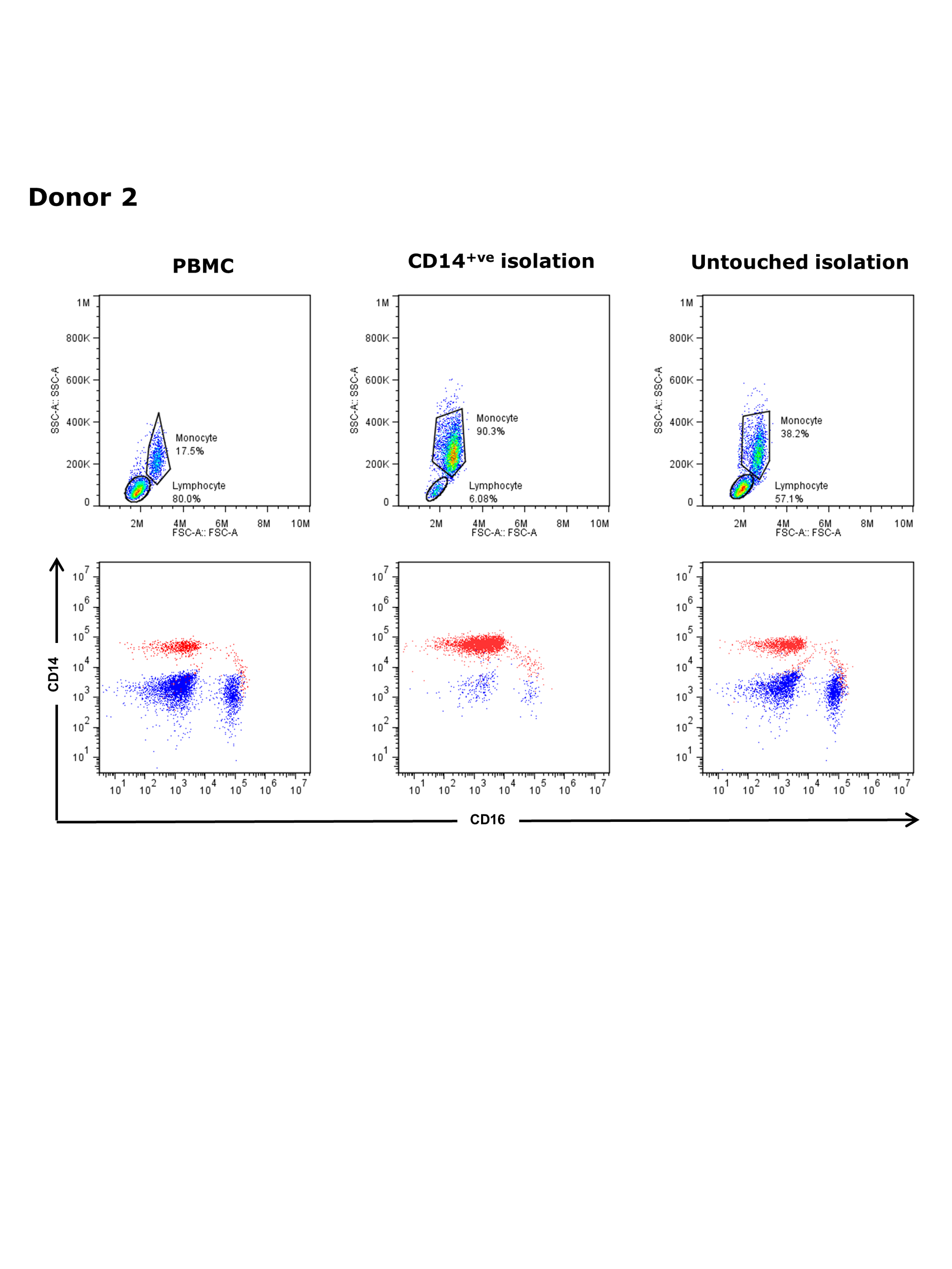

Supplement: S4 Fig — Data shows single cell data dot plots for SSC/FSC revealing the monocyte and lymphocyte gates. These were then compared for CD14 and CD16 expression to show the different monocyte subsets (red) and lymphocytes (blue) present. Note the particularly low yield of monocytes at only 38% in the untouched harvest. The CD16 high (blue cells) are NK cells present in the lymphocyte gate. (TIF) [file pone.0180267.s004.tif]

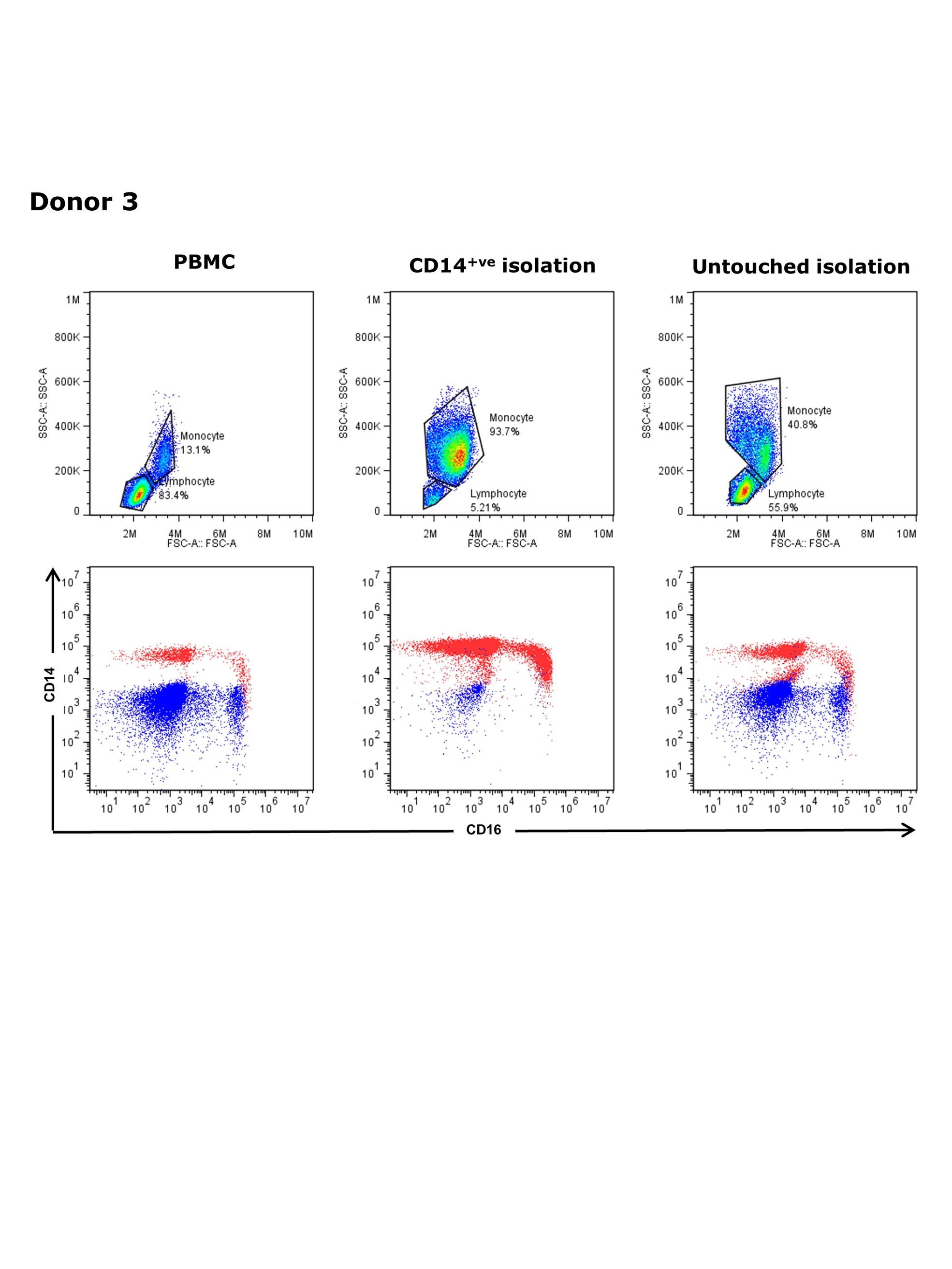

Supplement: S5 Fig — Data shows single cell data dot plots for SSC/FSC revealing the monocyte and lymphocyte gates. These were then compared for CD14 and CD16 expression to show the different monocyte subsets (red) and lymphocytes (blue) present. Note the low yield of monocytes at only 40.8% in the untouched harvest. The CD16 high (blue cells) are NK cells present in the lymphocyte gate. (TIF) [file pone.0180267.s005.tif]

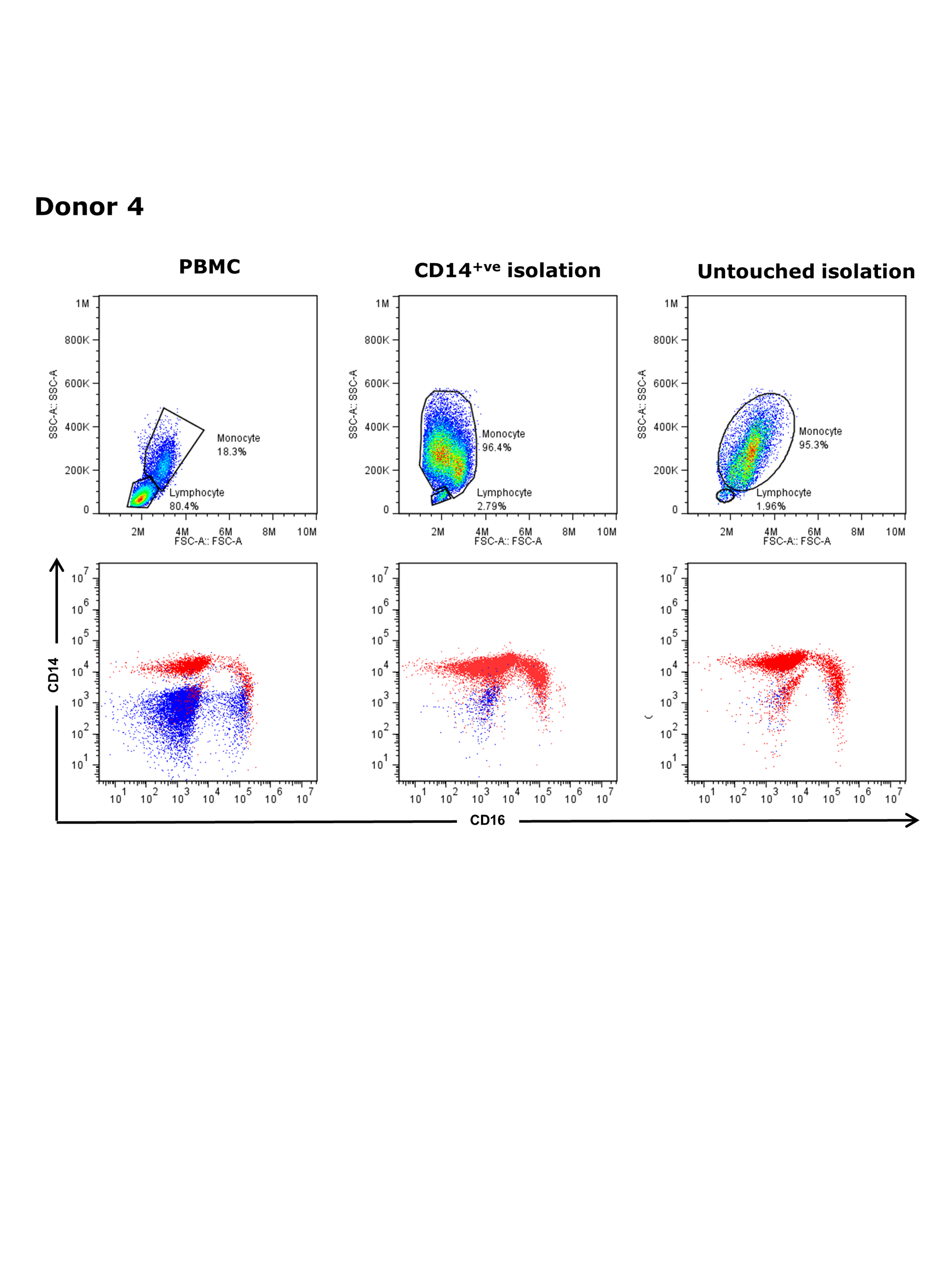

Supplement: S6 Fig — Data shows single cell data dot plots for SSC/FSC revealing the monocyte and lymphocyte gates. These were then compared for CD14 and CD16 expression to show the different monocyte subsets (red) and lymphocytes (blue) present. This was the only donor to produce an untouched monocyte yield above 90%. Note the lack of NK cells post isolation for this donor. (TIF) [file pone.0180267.s006.tif]

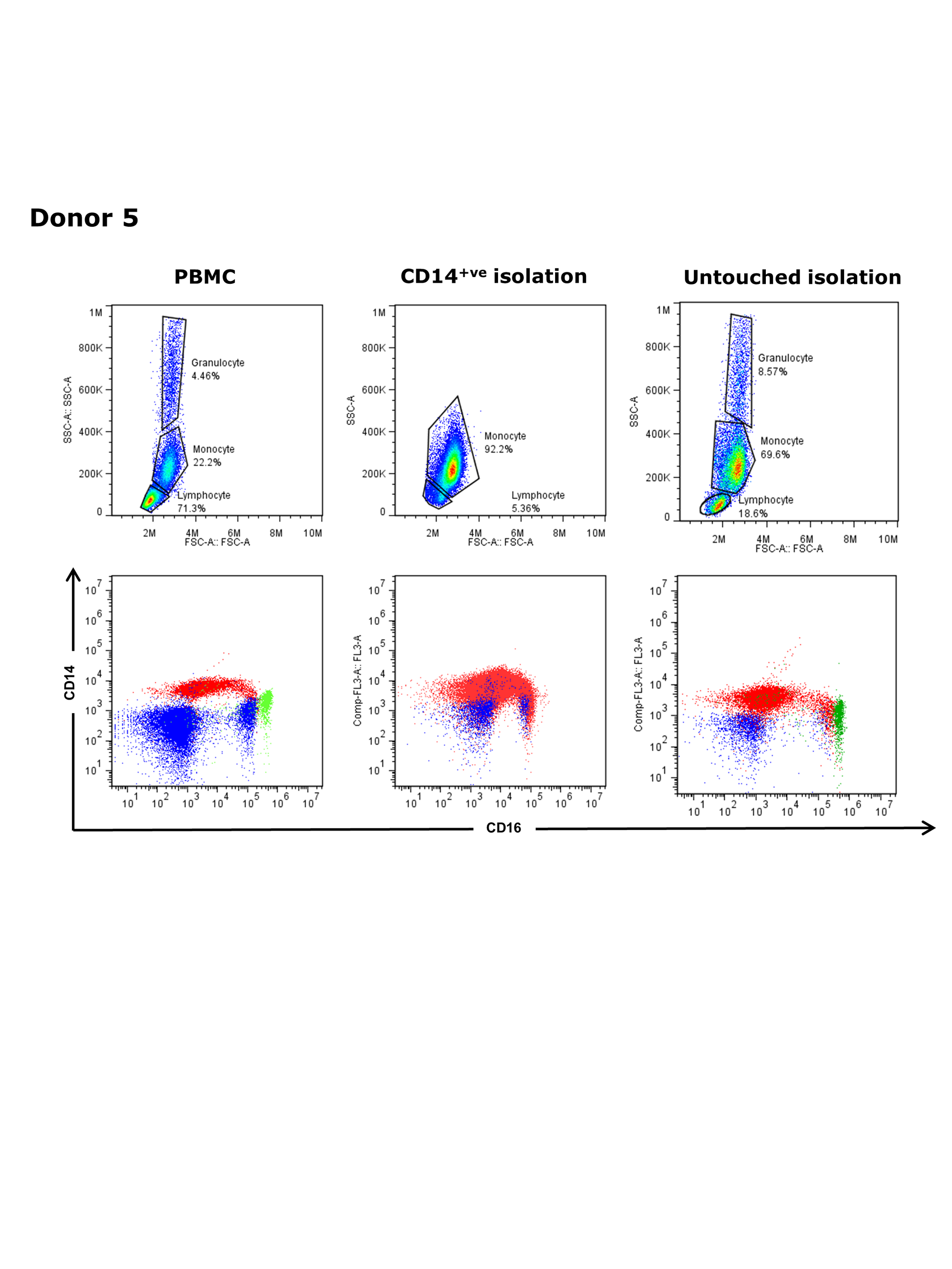

Supplement: S7 Fig — Data shows single cell data dot plots for SSC/FSC revealing the monocyte, lymphocyte and presence of a granulocyte gate. These were then compared for CD14 and CD16 expression to show the different monocyte subsets (red), lymphocytes (blue) and granulocytes (green). The CD16hi (blue cells) are NK cells present in the lymphocyte gate and the CD16hi (green cells) are neutrophils. The presence of the neutrophils was of concern in the untouched fraction, however, this could not be predicted as the antibody cocktail for the untouched assay is not made available to users. (TIF) [file pone.0180267.s007.tif]
