# Supplementary material for: ECIS technology reveals that monocytes isolated by CD14+ve selection mediate greater loss of BBB integrity than untouched monocytes, which occurs to a greater extent with IL-1β activated endothelium in comparison to TNFα
Source: PLoS One. 2017 Jul 21;12(7):e0180267. doi: 10.1371/journal.pone.0180267 (PMC5521748; doi:10.1371/journal.pone.0180267)
Supplement: S3 Data — The data file for S3 Data is located at Figshare’s online digital repository at https://figshare.com/s/8fd60608b49e782de618. (DOCX) [file pone.0180267.s010.docx]

The data file for S3 Data is located at Figshare’s online digital repository at: <https://figshare.com/s/8fd60608b49e782de618>
